# A Stepped Gate Oxide Structure for Suppressing Gate-Induced Drain Leakage in Fully Depleted Germanium-on-Insulator Multi-Subchannel Tunneling Field-Effect Transistors

**DOI:** 10.3390/mi16040375

**Published:** 2025-03-26

**Authors:** Rui Chen, Liming Wang, Ruizhe Han, Keqin Liao, Xinlong Shi, Peijian Zhang, Huiyong Hu

**Affiliations:** 1 Laboratory of Analog Integrated Circuits, Hangzhou Institute of Technology, Xidian University, Hangzhou 311231, China; 21111110114@stu.xidian.edu.cn (R.C.); 20009101593@stu.xidian.edu.cn (R.H.); 24251215542@stu.xidian.edu.cn (K.L.); xinlong.shi@stu.xidian.edu.cn (X.S.); huhy@xidian.edu.cn (H.H.); 2 National Laboratory of Analog Integrated Circuits, Chongqing 400060, China

**Keywords:** FD-GeOI, TFET, GIDL, multi-subchannel, stepped gate oxide

## Abstract

To address the severe gate-induced drain leakage (GIDL) issue in fully depleted germanium-on-insulator (FD-GeOI) multi-subchannel tunneling field-effect transistors (MS TFETs), this paper proposes a stepped gate oxide (SGO) structure. In the off-state, the SGO structure effectively suppresses GIDL by reducing the electric field intensity at the channel/drain interface while simultaneously decreasing gate capacitance to reduce static power consumption. Based on an accurate device model, a systematic investigation was conducted into the effects of varying the thickness and length of the SGO structure on TFET performance, enabling the optimization of the SGO design. The simulation results demonstrate that, compared to normal MS TFETs, the SGO MS TFET reduces the off-state GIDL current ($I_{\mathrm{off}}$) from $4.6\times 10^{-7}$ A to $2.6\times 10^{-11}$ A, achieving a maximum improvement of 4.22 orders of magnitude in the on-state-to-off-state current ratio ($I_{\mathrm{on}}/I_{\mathrm{off}}$) and a 28% reduction in subthreshold swing (SS). Furthermore, compared to lightly doped drain (LDD) MS TFETs, the SGO MS TFET achieves a 32% reduction in total gate capacitance and a 23% enhancement in carrier mobility at the channel/drain interface. This study demonstrates that SGO provides an effective solution for GIDL suppression.

## 1. Introduction

Conventional TFETs exhibit advantages such as a low off-state current [1], minimal static power consumption [2], and a steep subthreshold swing [3]. However, their limited on-state current significantly restricts their practical application [4]. Fully depleted (FD) Multi-Subchannel TFETs (MS TFETs) address this limitation by modifying the device architecture [5,6]. Unlike traditional TFETs, MS TFETs maintain a uniform doping concentration in both the channel and drain regions, while the source region is oppositely doped [7]. A high-work-function metal is used as the gate electrode, ensuring complete channel depletion at zero gate bias ($V_{GS}$ = 0 V) [8]. In the on-state, the depletion region disappears, and the channel transitions to an $n^{+}$ state, with significant tunneling occurring across the entire contact interface of the $p^{+}n^{+}$ junction formed between the source and channel regions. This generates a larger tunneling current. Unlike the inversion-mode operation of conventional TFETs, the fully depleted operational mechanism of the MS TFET enables it to function in flatband mode or accumulation mode. The MS TFET turns off the device by depleting the heavily doped channel through the metal gate. This allows the channel and drain regions to be simultaneously doped with $n^{+}$ through identical ion implantation doses, eliminating the need for the additional masking steps required for distinct doping of the drain and channel in conventional TFETs. Consequently, the MS TFET achieves reduced photolithographic complexity, offering a simplified fabrication process, an enhanced on-state current ($I_{on}$), and superior drive capability, making it a promising candidate for future electronic applications.

To ensure full channel depletion at zero bias, a thin channel and a gate electrode with a large work function are preferred. However, this design induces significant band-to-band tunneling (BTBT) at the channel/drain interface in the off-state, leading to severe GIDL and increased off-state current ($I_{off}$) [9,10,11]. To mitigate the gate-induced drain leakage (GIDL) in MS TFETs, two architectural approaches based on fully depleted silicon-on-insulator (FD-SOI) [12,13] substrates have been proposed in previous studies: one integrating a field plate (FP) [14,15] and another employing a lightly doped drain (LDD) structure [16,17,18]. Although the FP configuration demonstrates enhanced device performance, its implementation necessitates an extra metal type that introduces additional process complexity. While the LDD design proves effective in GIDL suppression, this advantage comes at the expense of elevated on-state resistance, consequently degrading the drive current [19,20].

To address the challenges of GIDL suppression and the optimization of device performance in MS TFETs, this study proposes a stepped gate oxide (SGO) MS TFET based on fully depleted germanium-on-insulator (FD-GeOI). The SGO structure effectively mitigates the leakage tunneling effect caused by the large metal work function through electric field modulation at the drain/channel interface during the off-state, thereby significantly reducing GIDL current. Systematic simulation studies demonstrate that both the $I_{on}/I_{\mathrm{off}}$ ratio and SS of the SGO MS TFET are improved compared to normal MS TFETs. Subsequently, we analyze and optimize the dimensions of the suppression oxide in SGO (suppression oxide Thickness ($T_{S-\mathrm{oxide}}$)/suppression oxide Length ($L_{S-\mathrm{oxide}}$) = 3–15 nm/0–25 nm). The simulation results reveal that the device achieves optimal performance when $T_{S-\mathrm{oxide}}$ = 12 nm and $L_{S-\mathrm{oxide}}$ = 20 nm. Compared to normal MS TFETs, the SGO MS TFET demonstrates (1) a reduction in $I_{off}$ from $4.6\times 10^{-7}$ A to $2.6\times 10^{-11}$ A, achieving a decrease of 4.25 orders of magnitude; (2) a 28% reduction in SS; and (3) an improvement in the $I_{on}/I_{\mathrm{off}}$ ratio by 4.22 orders of magnitude. Furthermore, compared to LDD, SGO exhibits a 32% reduction in total gate capacitance, a 23% increase in the mobility of the drain carrier, and a higher electron current density.

## 2. Device Structure and Simulation Method

### 2.1. Device Structure

**Figure 1:**
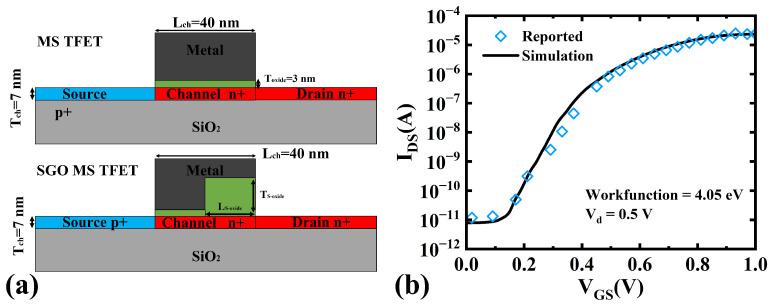
(**a**) Comparison of device structures for normal MS TFET and SGO MS TFET. (**b**) Validation of simulation setup by reproducing results of [32].

Figure 1a shows the cross-section of a standard FD-GeOI MS TFET and the SGO MS TFET. In the SGO MS TFET, the gate oxide is composed of two layers, control oxide and suppression oxide, in contrast to the standard MS TFET. The control oxide near the source region turns the device off when $V_{GS}$ is 0 V, similarly to the normal MS TFET. The suppression oxide near the drain reduces the GIDL between the channel and drain. As $V_{GS}$ increases, the depletion region under the suppression oxide disappears first, followed by the depletion region beneath the control oxide, which turns the device on.

### 2.2. Simulation Methodology

In the simulations, the suppression oxide was set to a length of 20 nm and a thickness of 12 nm, while the control oxide and channel thicknesses were 3 nm and 7 nm, respectively. Both the normal MS TFET and SGO MS TFET featured a uniformly doped channel ($1\times 10^{19}\;{\mathrm{cm}}^{-3}$). A TiN gate electrode with a work function of 4.5 eV was used to ensure full channel depletion in the off-state (as shown in Table 1). HfO_2_, a high-k material, served as the gate dielectric for both the control and suppression oxides (as shown in Table 1). Simulations were carried out using Sentaurus TCAD. The selection of device models was performed by using the Advanced Calibration User Guide [21]. The dynamic nonlocal BTBT model [22] was considered for the band-to-band tunneling of charge carriers between the drain and channel. Calibration of the dynamic nonlocal BTBT model parameters was based on experimental results from the literature [23,24,25,26]. The parameters of germanium were A = 1.46 × 10^17^ cm^−3^ s^−1^ and B = 3.59 × 10^6^ V/cm. Due to the high doping level, the BGN model [27] was also included, because the effective bandgap directly influences the tunneling current. Also, the SRH recombination model [28] was included due to the presence of a high impurity atom concentration in the channel, and Fermi–Dirac statistics [29] were included to calculate the intrinsic carrier concentration. For more accurate current calculations, the field-dependent [30] and doping-dependent mobility degradation model and drift-diffusion current transport model [31] were also considered. The simulation settings were calibrated by reproducing the results from [32], as shown in Figure 1b.

### 2.3. Suggested Process Flow

**Figure 2:**
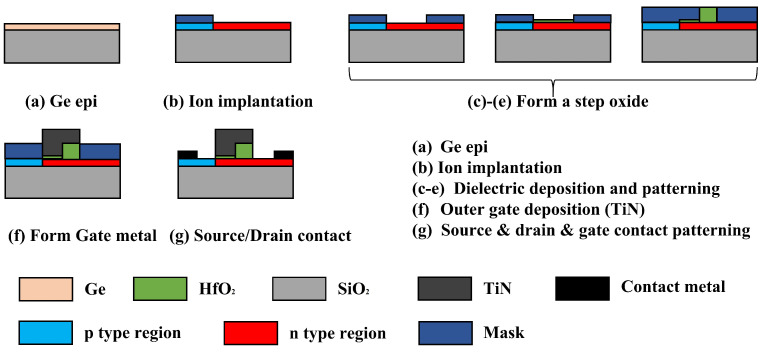
Suggested fabrication flow of SGO MS TFET.

As shown in Figure 2, SGO device fabrication involves the following key steps: hydrogen ion implantation, wafer bonding, layer transfer, and surface treatment form a high-quality single-crystal Ge layer on SiO_2_ [33]. Low-temperature epitaxial growth minimizes lattice defects caused by Ge-Si mismatch. The source, channel, and drain regions are doped via ion implantation (p-type boron, n-type phosphorus), followed by rapid thermal annealing (RTA) [34] for dopant activation. The gate dielectric process uses atomic layer deposition (ALD) [35] with HfCl_4_ and H_2_O precursors to deposit a 3 nm thick HfO_2_ layer at 0.15 nm/cycle. Self-aligned double patterning (SADP) [36] defines regions requiring thicker dielectrics, where an additional 9 nm of HfO_2_ is deposited via 70 ALD cycles at 0.13 nm/cycle. In situ ellipsometry ensures precise thickness control (total 12 nm). CF_4_/Ar plasma etching removes the SiC hard mask with controlled surface roughness (Ra < 0.3 nm), followed by spike annealing (SPA, 1 s at 1000 °C) to repair interface defects [37]. Finally, a conformal TiN metal gate is formed via ALD, and vertical contact holes are created using Cl_2_/BCl_3_ plasma etching with a bottom anti-reflective coating (BARC) for enhanced pattern fidelity [38,39,40].

In contrast, LDD MS TFET fabrication requires additional photolithography and doping steps. The channel region is defined through photolithography and heavy phosphorus (P) doping, while the drain region involves two-step photolithography: (1) Low-energy P implantation forms an $n^{-}$ LDD region near the channel to mitigate the electric field concentration. (2) High-dose arsenic (As) implantation defines the $n^{++}$ drain contact region after precise alignment, ensuring low series resistance [41]. A uniform 3 nm HfO_2_ gate dielectric layer is grown via ALD. Compared to SGO, which integrates the drain and channel regions in a single photolithography step, the LDD approach requires multiple doping steps and photolithography cycles, and strict alignment between the $n^{+}$ channel and $n^{-}$ lightly doped regions. This increases process complexity, particularly in doping control and patterning precision.

## 3. Results and Discussion

The introduction of the SGO structure in the FD-GeOI MS TFET significantly influences key performance metrics, including the $I_{off}$, $I_{on}$/$I_{off}$ ratio and SS. The SGO design effectively modulates the electric field distribution within the device, leading to notable improvements in these parameters.

### 3.1. Off-State Current Reduction

**Figure 3:**
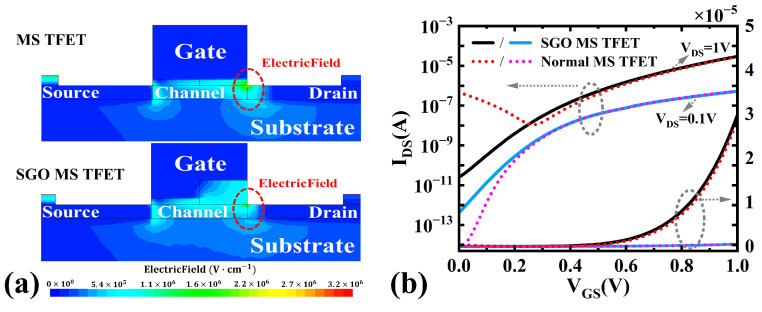
(**a**) The off-state electric fields and (**b**) transfer characteristics of the normal MS TFET and SGO MS TFET.

In MS TFETs, high gate metal work function is required to deplete the heavily doped channel, which inevitably leads to the generation of GIDL. This leakage current in the off-state contributes to increased static power dissipation, degrading both the power efficiency and overall device performance. To mitigate this issue, an SGO configuration is introduced, which effectively weakens the gate/drain electric field intensity in the off-state (as shown in Figure 3a), thereby suppressing GIDL. Figure 3b illustrates the transfer characteristics comparison between the normal MS TFET and those employing the SGO structure. With the implementation of the SGO design, $I_{\mathrm{off}}$ is reduced from 4.6 × $10^{-7}$ A to 2.6 × $10^{-11}$ A, representing an improvement of 4.25 orders of magnitude compared to the normal MS TFET. The SGO structure exhibits a negligible impact on $I_{on}$, thereby maintaining the drive current at approximately 3.0 × $10^{-5}$ A.

**Figure 4:**
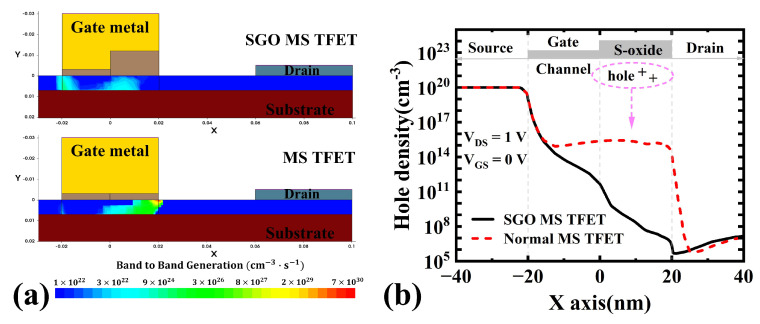
(**a**) Band-to-band generation and (**b**) surface hole concentration of normal MS TFET and SGO MS TFET at $V_{GS}$ = 0 V and $V_{DS}$ = 1 V.

**Figure 5:**
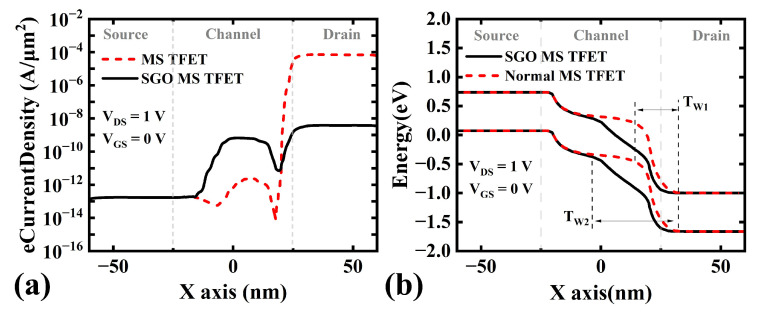
(**a**) eCurrentDensity and (**b**) surface band diagram of normal MS TFET and SGO MS TFET at $V_{GS}$ = 0 V and $V_{DS}$ = 1 V.

With the introduction of a thicker suppression oxide in the SGO structure, the gate’s depletion effect on channel electrons weakens, reducing the number of inversion holes on the channel’s upper surface (see Figure 4b). Consequently, the built-in electric field of the pn junction and BTBT decrease. At the gate/drain junction, the off-state tunneling probability and current density are significantly lower than those in the normal MS TFET (see Figure 4a and Figure 5a). From an energy band perspective, the thicker suppression oxide increases the tunneling barrier width $T_{W_{1/2}}$ (see Figure 5b), further reducing the carrier tunneling probability. Thus, the SGO structure effectively suppresses the GIDL effect caused by a large gate metal work function.

### 3.2. Analysis of SGO Structural Parameters

The performance of the SGO MS TFET strongly depends on the thickness and length of the suppression oxide. Optimizing these parameters can simultaneously improve the $I_{on}$/$I_{off}$ ratio and SS, further enhancing overall device performance.

#### 3.2.1. Effect of Suppression Oxide Thickness

**Figure 6:**
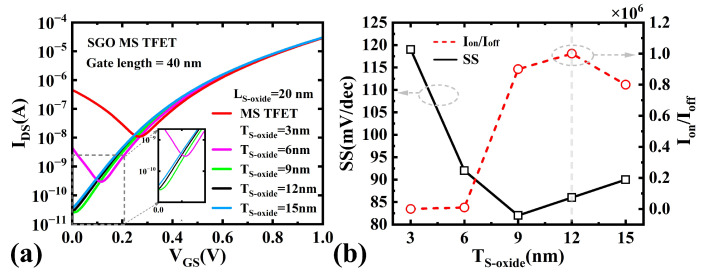
(**a**) $I_{DS}$-$V_{GS}$ for Different $T_{S-oxide}$ Values. (**b**) Variation of SS and $I_{on}$/$I_{off}$ Ratio with $T_{S-oxide}$ in SGO MS TFETs.

**Figure 7:**
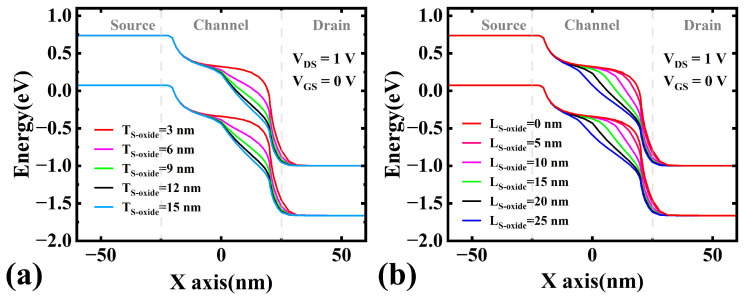
Energy band diagrams corresponding to different (**a**) $T_{S-oxide}$ and (**b**) $L_{S-oxide}$ values.

To elucidate the impact of the SGO structure on $I_{off}$, we simulated the variations in the $I_{DS}-V_{GS}$ characteristics, $I_{on}/I_{off}$ ratio, and SS under different suppression oxide thicknesses ($T_{S-oxide}$), as shown in Figure 6. The results indicate that as $T_{S-oxide}$ increases from 3 nm to 12 nm, $I_{off}$ of the SGO MS TFET decreases significantly from $4.6\times 10^{-7}$ A to $2.6\times 10^{-11}$ A, SS improves from 119 mV/dec to 86 mV/dec, and the $I_{on}/I_{off}$ ratio increases substantially from $0.6\times 10^{2}$ to $1\times 10^{6}$. This improvement can be attributed to two factors: First, the increased $T_{S-oxide}$ (from 3 nm to 12 nm) effectively reduces the electric field intensity at the channel-drain junction in the off-state; second, it ensures the complete depletion of the heavily doped channel while slightly increasing the electron concentration in the channel (still below the neutral electron concentration). Under the combined effects of reduced electric field intensity and increased channel electron concentration, the energy band at the gate/drain interface becomes progressively flatter, preventing valence band electrons from tunneling (see Figure 7a). Consequently, $I_{off}$ gradually decreases, while the $I_{on}/I_{off}$ ratio and SS are further optimized, leading to a significant enhancement in device performance.

However, as the suppression oxide thickness continues to increase, the thickened oxide layer gradually weakens the gate’s ability to control channel depletion near the drain side. Therefore, when $T_{S-oxide}$ is further increased from 12 nm to 15 nm, the gate metal’s ability to deplete electrons in the drain-side channel is further compromised. At this point, the heavily doped channel fails to be fully depleted, resulting in an elevated electron concentration in the channel (exceeding the neutral electron concentration). This implies that the effective channel region that can be depleted by the gate shortens, making the device more susceptible to short-channel effects (SCEs), which increases $I_{off}$, SS and degrades the $I_{on}/I_{off}$ ratio. As shown in Figure 6, when $T_{S-oxide}=15$ nm, $I_{off}$ increases from $2.6\times 10^{-11}$ A to $3.5\times 10^{-11}$ A, the $I_{on}/I_{off}$ ratio drops from $1.0\times 10^{6}$ to $0.8\times 10^{6}$, and SS deteriorates from 86 mV/dec to 90 mV/dec.

In summary, as the suppression oxide thickness increases, the gate’s control over the channel in this region continuously weakens until it is eventually lost, causing the device performance to first improve and then degrade. Specifically, when $T_{S-oxide}$ is 12 nm, GIDL is completely suppressed, and the channel is in a critical depletion state, achieving optimal device performance. However, when the thickness is further increased to 15 nm, the channel is no longer fully depleted, triggering SCEs and resulting in degraded device performance.

#### 3.2.2. Effect of Suppression Oxide Length

**Figure 8:**
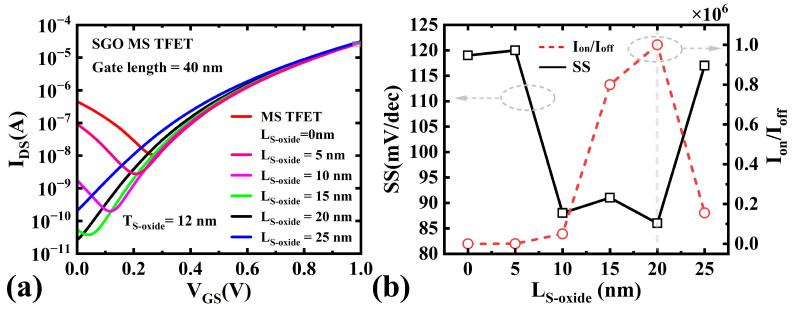
(**a**) $I_{DS}$-$V_{GS}$ for Different $L_{S-oxide}$ Values. (**b**) Variation of SS and $I_{on}$/$I_{off}$ Ratio with $L_{S-oxide}$ in SGO MS TFETs.

We simulated the transfer characteristics, $I_{on}/I_{off}$ ratio, and SS for different suppression oxide lengths ($L_{S-oxide}$), as shown in Figure 8. As $L_{S-oxide}$ increases from 0 nm to 20 nm, $I_{off}$ of the TFET decreases significantly from $4.6\times 10^{-7}$ A to $2.6\times 10^{-11}$ A, while SS improves from 119 mV/dec to 86 mV/dec, and the and $I_{on}/I_{off}$ ratio increases substantially from $0.6\times 10^{2}$ to $1.0\times 10^{6}$. This improvement can be attributed to the following mechanisms: As $L_{S-oxide}$ increases, the hole charge region beneath the oxide decreases significantly, and the electric field near the drain side of the gate is also significantly reduced. These changes cause the energy band bending near the drain side of the channel to flatten during the off-state (see Figure 7b), increasing the effective tunneling barrier width and thereby significantly reducing the tunneling probability and leakage current in this region. However, when $L_{S-oxide}$ is further increased (>20 nm), the effective channel depletion region gradually shrinks, leading to the aggravation of SCEs and an increase in $I_{off}$. As shown in Figure 8, when $L_{S-oxide}$ increases from 20 nm to 25 nm, $I_{off}$ rises from $2.6\times 10^{-11}$ A to $2.0\times 10^{-10}$ A, the $I_{on}/I_{off}$ ratio drops from $1\times 10^{6}$ to $1.6\times 10^{5}$, and SS deteriorates from 86 mV/dec to 117 mV/dec. This indicates that an excessively long suppression oxide layer weakens the gate’s control over the channel, thereby degrading device performance.

As the gate voltage gradually increases, the channel region corresponding to the thicker suppression oxide layer (with weaker gate control capability) exits the depletion state first, while the channel region associated with the thinner control oxide layer (with stronger gate control capability) exits the depletion state last. Regardless of the variations in the thickness of the suppression oxide layer, when the gate voltage continues to increase, the electron concentration in the channel region under the control oxide layer eventually recovers to the doping concentration. At this point, the pn junction at the source/channel interface of devices with different suppression oxide thicknesses operates under the same doping concentration and reverse bias ($V_{DS}$), producing identical quantum tunneling. Therefore, $I_{on}$ of the MS TFET is largely unaffected by the SGO structure.

Based on the analysis of the aforementioned results, the parameters of the suppression oxide in the SGO structure are optimized to their ideal values, with a $T_{S-oxide}$ of 12 nm and a $L_{S-oxide}$ of 20 nm. Compared to the normal MS TFET, the SGO MS TFET demonstrates superior performance metrics. The introduction of the SGO structure leads to a significant reduction in $I_{off}$ and an improvement in the $I_{on}$/$I_{off}$ ratio by 4.22 orders of magnitude. Additionally, the SS is improved to 86 mV/dec, indicating more efficient switching behavior (as shown in Table 2).

### 3.3. Capacitance and Mobility Analysis

After determining the optimal suppression oxide dimensions ($T_{S-oxide}=12$ nm and $L_{S-oxide}=20$ nm), a C-V characteristic study was conducted for the SGO MS TFET, normal MS TFET, and LDD MS TFET to evaluate the impact of the SGO structure on circuit-level device performance. First, the total gate capacitance ($C_{gg}$) was analyzed by computing the frequency-dependent admittance matrix. The total gate capacitance of the TFETs can be expressed using Equation (1), where $C_{s}$ represents the depletion capacitance given by $\varepsilon_{Si}/T_{ch}$, and $C_{ox}$ denotes the gate oxide capacitance, which can be derived from Equation (2). According to Equations (1) and (2), an increase in $T_{S-oxide}$ leads to a reduction in $C_{gg}$, thereby minimizing gate capacitance and improving device performance [42].

(1) $$\frac{1}{C_{gg}}=\frac{1}{C_{ox}}+\frac{1}{C_{s}}$$

(2) $$C_{ox}=C_{ox1}+C_{ox2}=\frac{\varepsilon_{ox}}{T_{Coxide}}+\frac{\varepsilon_{ox}}{T_{Toxide}}$$

**Figure 9:**
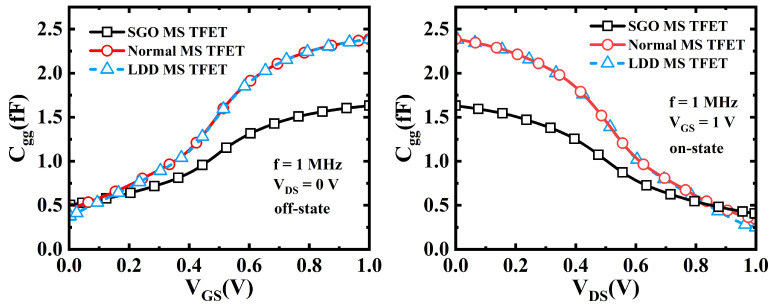
(**a**) Off-state and (**b**) on-state total gate capacitance of SGO MS TFET, normal MS TFET, and LDD MS TFET.

Figure 9 compares the $C_{gg}$ of the SGO MS TFET, normal MS TFET, and LDD MS TFET under different bias conditions. From Figure 9a ($C_{gg}$ vs. $V_{GS}$ at $V_{DS}$ = 0 V), it is observed that the SGO MS TFET exhibits the lowest $C_{gg}$, whereas the LDD MS TFET and normal MS TFET have similar and higher capacitance values. From Figure 9b ($C_{gg}$ vs. $V_{DS}$ at $V_{GS}$ = 1 V), the $C_{gg}$ of the SGO MS TFET decreases more gradually as $V_{DS}$ increases, while the normal MS TFET and LDD MS TFET maintain relatively higher capacitance levels. This phenomenon occurs because the SGO structure effectively reduces the gate/drain electric field, minimizing edge capacitance effects and thereby lowering parasitic capacitance. In contrast, the LDD MS TFET, due to its lightly doped drain, suppresses GIDL but does not significantly reduce $C_{gg}$. As a result, its capacitance characteristics remain similar to those of the normal MS TFET, leading to persistent parasitic effects.

Compared to the normal MS TFET and LDD MS TFET, the $C_{gg}$ of the SGO MS TFET is reduced by 32%. Due to its lower gate capacitance, the SGO MS TFET can minimize parasitic capacitance effects, thereby improving switching speed in high-frequency applications.

**Figure 10:**
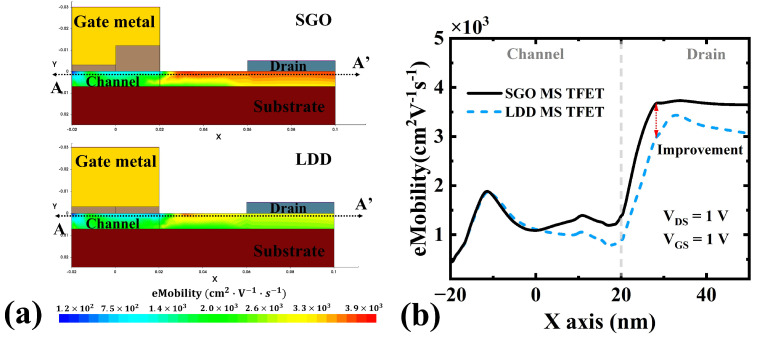
(**a**) Carrier mobility (eMobility) of the SGO MS TFET and LDD MS TFET. (**b**) Carrier mobility (eMobility) at the A-A’ cross-section.

**Figure 11:**
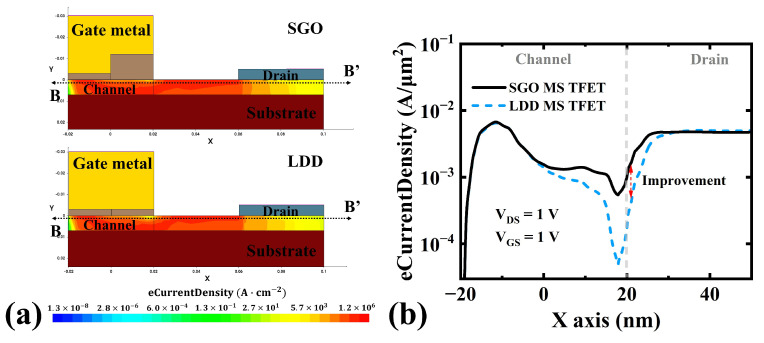
(**a**) Electron current density (eCurrentDensity) of the SGO MS TFET and LDD MS TFET. (**b**) Electron current density (eCurrentDensity) at the B-B’ cross-section.

Figure 10 and Figure 11 compare the carrier mobility [43,44] and electron current density [45,46] in the SGO MS TFET and LDD MS TFET. These figures provide further insight into the SGO structure’s impact on carrier transport and overall device performance. The SGO MS TFET exhibits higher carrier mobility (increased by 23%) near the channel/drain interface compared to the LDD MS TFET. This improvement is due to the SGO reducing the vertical electric field (superior in reducing electric field intensity/peaks compared to LDD), which mitigates mobility degradation caused by interface roughness and scattering. Higher mobility contributes to enhanced carrier transport efficiency, leading to improved on-state performance without additional power consumption.

In addition, the electron current density of the SGO MS TFET is significantly higher than that of the LDD MS TFET (increased by 2.9 times). This advantage is mainly attributed to the higher carrier concentration and mobility achieved near the drain side, demonstrating superior carrier transport capability. The SGO structure, through optimized design of the suppression oxide layer, not only ensures a higher electron current density but also maintains high carrier mobility. In contrast, although the LDD structure can mitigate the GIDL effect to some extent, its lower doping concentration significantly increases the parasitic resistance at the drain, thereby limiting the improvement in electron current density. Therefore, compared to the LDD MS TFET, the SGO structure exhibits superior performance in both carrier mobility and electron current density, thereby demonstrating greater overall advantages in switching applications.

## 4. Conclusions

This study addresses the issue of GIDL in FD-GeOI MS TFETs by proposing an innovative SGO structure. Compared to the traditional LDD structure, the SGO design is simpler to fabricate and significantly enhances device performance through electric field modulation at the channel/drain junction. By systematically investigating different SGO dimensions, including thickness and length, we determined the optimal SGO configuration that minimizes $I_{off}$, optimizes SS, and maximizes the $I_{on}/I_{off}$ ratio. The simulation results demonstrate that the optimized SGO structure reduces $I_{off}$ from $4.6\times 10^{-7}$ A to $2.6\times 10^{-11}$ A, attributed to the expansion of the tunneling barrier width, representing a significant improvement over normal MS TFETs. Additionally, compared to LDD MS TFETs, the SGO MS TFET reduces $C_{gg}$ by 32% through increased suppression oxide thickness. The SGO also exhibits superior carrier transport characteristics, with the electron current density being significantly higher than that of LDD (increased by 2.9 times), and drain carrier mobility improved by 23%. This enhancement in mobility ensures low parasitic resistance while improving overall device performance. Energy band engineering achieved through SGO modulation further optimizes the SS of the MS TFET, reducing it from 119 mV/dec to 86 mV/dec—a 28% improvement. Notably, these enhancements are achieved without compromising $I_{on}$ while maintaining a high $I_{on}/I_{off}$ ratio. The SGO fabrication process is fully compatible with existing semiconductor manufacturing technologies, providing a scalable and novel solution for GIDL suppression in both 2D and 3D FD-GeOI devices. Its performance and scalability surpass those of LDD.

## Figures and Tables

**Table 1:** Device structure parameters.

Parameter	Normal MS TFET	SGO MS TFET
Gate length ($L_{ch}$)	40 nm	40 nm
Suppression oxide thickness ($T_{S-\mathrm{oxide}}$)	-	3–15 nm
Suppression oxide length ($L_{S-\mathrm{oxide}}$)	-	0–25 nm
Gate work function	4.5 eV	4.5 eV
Source doping	1 × $10^{20}$/cm^3^	1 × $10^{20}$/cm^3^
Drain/channel doping	1 × $10^{19}$/cm^3^	1 × $10^{19}$/cm^3^
Device layer thickness ($T_{ch}$)	7 nm	7 nm
Control oxide thickness ($T_{C-\mathrm{oxide}}$)	3 nm	3 nm

**Table 2:** Performance comparison between conventional MS TFET and SGO MS TFET.

Parameter	Normal MS TFET	SGO TFET	Improvement
$I_{off}$ (A)	4.6 × $10^{-7}$	2.6 × $10^{-11}$	Reduced by 4.25 orders of magnitude
$I_{on}$ (A)	2.9 × $10^{-5}$	3.0 × $10^{-5}$	No significant loss
$I_{on}$/$I_{off}$ Ratio	0.6 × $10^{2}$	1 × $10^{6}$	Increased by 4.22 orders of magnitude
SS (mV/dec)	119	86	Reduced by 28%

## Data Availability

The original contributions presented in this study are included in the article. Further inquiries can be directed to the corresponding author.

## Abbreviations

The following abbreviations are used in this manuscript:

FD-SOI	Fully depleted Silicon-on-Insulator
FD-GeOI	Fully depleted Germanium-on-Insulator
GIDL	Gate-induced drain leakage
SGO	Stepped gate oxide
MS TFET	Multi-subchannel Tunneling field effect transistor
LDD	Lightly doped drain
FP	Field plate
$I_{on}$	On-state current
$I_{off}$	Off-state current
$I_{on}$/$I_{\mathrm{off}}$	The on-state to off-state current ratio
SS	Subthreshold swing
BTBT	Band-to-band tunneling
BGN	Bandgap narrowing
SRH	Shockley–Read–Hall
RTA	Rapid thermal annealing
ALD	Atomic layer deposition
SADP	Self-aligned double patterning
SPA	Spike annealing
BARC	Bottom anti-reflective coating
P	Phosphorus
As	Arsenic
C*_gg_*	Total gate capacitance
